# Diurnal patterns of sedentary time in rheumatoid arthritis: associations with cardiovascular disease risk

**DOI:** 10.1136/rmdopen-2020-001216

**Published:** 2020-07-14

**Authors:** Sally A M Fenton, Nikos Ntoumanis, Joan L Duda, George S Metsios, Peter C Rouse, Chen-an Yu, George D Kitas, Jet J C S Veldhuijzen van Zanten

**Affiliations:** 1 School of Sport, Exercise and Rehabilitation Sciences, University of Birmingham, Birmingham, UK; 2 Russells Hall Hospital, Rheumatology, Dudley Group NHS Foundation Trust, Dudley, UK; 3 Physical Activity and Well-Being Research Group, School of Psychology, Curtin University, Perth, Australia; 4 Faculty of Education, Health and Wellbeing, University of Wolverhampton, Wolverhampton, UK; 5 Department for Health, University of Bath, Bath, UK; 6 Institute of Sport, University of Chichester, Chichester, UK

**Keywords:** Cardiovascular disease, psychology, rheumatoid arthritis, arthritis, rheumatoid, rehabilitation

## Abstract

**Objectives:**

Research demonstrates that sedentary behaviour may contribute towards cardiovascular disease (CVD) risk in rheumatoid arthritis (RA). This study explored diurnal patterns of sedentary time and physical activity (PA) in RA and examined associations with long-term CVD risk.

**Methods:**

97 RA patients wore an accelerometer for 7 days to assess sedentary time, light-intensity and moderate-to-vigorous-intensity PA. Estimated 10-year CVD risk was determined via QRISK score. Hourly estimates of sedentary time and PA (min/hour) were computed for valid-wear hours (ie, valid-wear = 60 min/hour of activity data, ≥3 days). Hourly data were averaged across time periods to represent morning (08:00–11:59), afternoon (12:00–17:59) and evening (18:00–22:59) behaviour. Participants providing data for ≥2 complete time periods/day (eg, morning/evening, or morning/afternoon) were used in the main analysis (n = 41). Mixed linear modelling explored the associations between 10-year CVD risk and within-person (time: morning, afternoon, evening) changes in sedentary time and PA.

**Results:**

Sedentary time was higher, and light-intensity and moderate-to-vigorous-intensity PA lower in the evening, compared to morning and afternoon. Significant interactions revealed individuals with higher CVD risk were more sedentary and did less light-intensity PA during the afternoon and evening. Findings remained significant after adjustment for disease duration, functional ability and erythrocyte sedimentation rate.

**Conclusion:**

Results suggest that the evening time period may offer a significant window of opportunity for interventions to reduce sedentary behaviour in RA and contribute to associated improvements in CVD risk. Due to inverse patterns of engagement, replacing sedentary time with light-intensity PA may offer an effective approach for intervention.

## INTRODUCTION

Sedentary behaviour is defined as any waking behaviour requiring ≤1.5 metabolic equivalents (METS), while undertaken in a sitting, lying or reclining posture).^1^ Prospective, epidemiological studies suggest sedentary behaviour (‘too much sitting’) associates with increased risk of cardiovascular disease (CVD) and is considered a modifiable risk factor for CVD morbidity and mortality.^2 3^

Rheumatoid arthritis (RA) is a chronic autoimmune disease, characterised by high-grade systemic inflammation and is associated with increased risk of CVD-related morbidity and mortality.^4 5^ Recent studies suggest that high levels of sedentary behaviour may also contribute to heightened CVD risk observed in this population.^6–8^ For example, cross-sectional studies have revealed significant positive associations between sedentary behaviour and estimated long-term CVD risk in RA^6 8^ as well as individual risk factors for CVD.^7 9^ In addition, research suggests it is not only the total volume of sedentary behaviour that may be detrimental for cardiovascular health in RA, but the manner in which it is accumulated.^6^ Specifically, a cross-sectional study reported that spending more time engaged in prolonged, uninterrupted periods of sedentary behaviour (sedentary bouts) is positively related to estimated long-term CVD risk in RA,^6^ a finding that has also been reported in epidemiological research.^10 11^

Research indicates that people living with RA spend approximately 60–70% of their waking hours sedentary,^6 12^ levels comparable to those observed in healthy adults.^12 13^ However, as people living with RA demonstrate a twoto threefold increased risk of CVD relative to adults in the general population,^5^ the impact of high levels of sedentary behaviour may be more severe for this patient group.^14^ Thus, interventions that target time spent sedentary, and particularly periods with prolonged sedentary bouts, may offer a great potential to improve cardiovascular health among people living with RA.^15^ However, while in recent years, our understanding of the role of sedentary behaviour for RA has advanced, we currently know very little about levels and patterns of sedentary time accumulation in this population. This information is critical to inform the development of successful interventions.

To date, the majority of studies examining sedentary behaviour in RA have provided a general indication of levels of habitual (daily) sedentary behaviour, reporting sedentary time according to the number of sedentary ‘minutes/day’.^15^ However, using an aggregate of sedentary time in this way (ie, collapsing measurements of sedentary time recorded across several days) masks the temporal patterns of this behaviour. That is, previous investigations do not offer a detailed account of specifically *when* people living with RA accumulate their sedentary time. Certainly, considering that diurnal fluctuations in disease symptoms (such as morning stiffness and fatigue) are reported, it is possible that there are also diurnal fluctuations in sedentary behaviour.

Research that examines time-based patterns of sedentary time accumulation in RA will therefore help to identify critical high sedentary time periods or ‘sedentary windows’, which may offer optimal opportunities for interventions to reduce sedentary behaviour in this particular population. Previous research has sought to examine diurnal patterns of sedentary time among older adults living without RA, with the intention of gathering information to inform interventions. These studies have reported significant differences between morning (eg, before 12:00–13:00), afternoon (eg, before 17:00–19:00) and evening (eg, after 17:00–19:00) sedentary behaviour, indicating sedentary time to peak in the evening after 17:00.^16–18^ These data have provided some insight into the patterns of sedentary time among older individuals, who—like people living with RA—may be at increased risk of the negative health consequences of sedentary behaviour. However, elucidating time-use patterns of sedentary behaviour in RA specifically is of particular importance, as factors related to the chronobiology of RA may impact diurnal patterns of sedentary time in a unique manner, which may hold implications for the design of behavioural interventions for this population (ie, to target ‘sedentary windows’). In addition, prior to advocating interventions that target periods of high sedentary, it is also essential to establish their potential for clinical efficacy among people living with RA. Studies that examine associations between diurnal patterns of sedentary time accumulation and pertinent health outcomes in RA (eg, CVD risk) are therefore also required.

It is equally important to study diurnal patterns of sedentary time in concurrence with other more active behaviours that represent the full breadth of the physical activity (PA) continuum (ie, light-intensity PA, moderate-to-vigorous intensity PA). Investigating sedentary time in isolation, without consideration of how it exists in the context of PA behaviours, may misrepresent what a sedentary lifestyle entails. Certainly, an understanding of the inter-relationship between sedentary time, light-intensity and moderate-to-vigorous-intensity PA is essential for the selection of appropriate (and effective) behavioural intervention targets. For example, owing to the strong, inverse correlation between sedentary time and light-intensity PA, it has been argued that encouraging light-intensity PA (eg, encompassing, standing, incidental movement, lifestyle-embedded activities of daily living) may help to reduce time spent sedentary among people living with RA, via a displacement effect.^6 9^

In order to accurately examine temporal patterns of sedentary time and PA, continuous monitoring of behaviour is required. Wearable accelerometers enable the collection of time-stamped movement data, permitting observation of chronological variability in levels of activity.^15^ Accelerometers also allow the more complex patterns of sedentary time accumulation to be investigated, such as the extent to which sedentary activity is accrued via engagement in prolonged (uninterrupted) sedentary bouts. Using data from the Physical Activity in Rheumatoid Arthritis (PARA), we have previously reported accelerometer-assessed overall *daily* sedentary time (minutes/day—based on the average of the accelerometer recordings of up to 7 days) and the length of prolonged, uninterrupted sedentary bouts (≥20 min), is linked to increased estimated 10-year risk of CVD in RA.^6^ In this investigation, we conduct an additional *hourly* analysis of PARA study data collected at baseline, in order to (1) examine the diurnal patterns of sedentary time (including uninterrupted sedentary bouts) and PA among people living with RA and (2) investigate whether these diurnal patterns of sedentary time and PA are associated with long-term CVD risk in these patients.

## METHODS

Patients with RA (n = 115) were recruited to the PARA study (Trial Number: ISRCTN04121489),^19^ from Rheumatology outpatient clinics at Russells Hall Hospital (RHH, Dudley Group of Hospitals NHS Trust, England). Interested patients were provided with study information sheets, and willing participants provided informed consent and were recruited. The local National Health Service Research Ethics Committee approved the study.

### Protocol

Participants attended two appointments 1 week apart to undertake assessments. During the first visit, a fasted blood sample was taken and a subsample of participants who consented to wear an accelerometer (n = 97) were fitted with a GT3X Actigraph accelerometer for the subsequent 7 days. Participants attended their second visit 1 week later to return accelerometers and undergo physical assessments to evaluate factors associated with their cardiovascular health.

### Measures

#### RA characteristics and medication

Disease activity was assessed via the disease activity score in 28 joints (DAS28) and erythrocyte sedimentation rate (ESR) determined using the Westergren method (Starrsed Compact, Mechatronics BV, Netherlands).^20^ *Functional disability* was measured using the Stanford Health Assessment Questionnaire (HAQ).^21^ *Disease duration* was self-reported, and current drug regime was recorded from patient medical notes (ie, use of Disease-Modifying Anti-Rheumatic Drugs, anti-Tumor Necrosis Factor therapy, non-steroidal anti-inflammatory drugs, analgesics, corticosteroids, cholesterol-lowering medication, medication for hypertension).

### Estimated (10-year) CVD risk

An estimate of 10-year CVD risk was determined by computing participants’ QRISK2 score,^22^ using participants’ age, gender, height and weight (body mass index (BMI)), blood pressure (systolic and diastolic), cholesterol (total/high-density lipoprotein (HDL) ratio), self-reported smoking status, diabetic status, presence of kidney disease and family history of heart disease.^23 24^^1^[1]QRISK2 as the QRISK3 was not available when primary data analysis for the PARA study was undertaken. QRISK2 has been used in all publications stemming from the PARA study to ensure consistency in the manner in which estimated long-term CVD risk is analysed and reported.
*Height* was measured to the nearest 0.5 cm using a standard height measure (Seca 214 Road Rod). *Weight* was determined using a Tanita BC 418MA Segmental Body Composition Analyser (Tanita Corporation, Tokyo, Japan). BMI was calculated from weight (kg) and height (m), as kg/m^2^. *Blood pressure* was assessed using an electronic sphygmomanometer (Datascope Accutor) as previously described.^23^ Fasted blood samples (≥ 12-hour fast) were collected for measurement of *total and HDL cholesterol*. Blood tests were carried out in the biochemistry laboratories at RHH, and serum levels of total cholesterol were analysed using the Vitros 5.1 chemistry system (www.orthoclinical.com). Smoking status, diabetic status, presence of kidney disease and family history were self-reported and corroborated with medical notes as appropriate.

### Sedentary time and physical activity

Sedentary time (total and bouts) and PA (light and moderate-to-vigorous) were assessed using GT3X accelerometers (Actigraph). Participants wore the accelerometer for 7 days during all waking hours, on the right hip, removing only for water-based activities (eg, swimming and bathing) and sleep. Accelerometers were initialised to collect data in 60-s epochs, and movement counts within each epoch were converted to activity counts (ie, counts per minute, cpm) to compute frequency, intensity and duration of activity behaviours. A 7 day wear protocol is reported to produce reliable estimates of sedentary time and PA, producing intraclass correlation coefficients ≥.80 across measurement days.^25 26^

### Accelerometer data reduction

**Figure 1:**
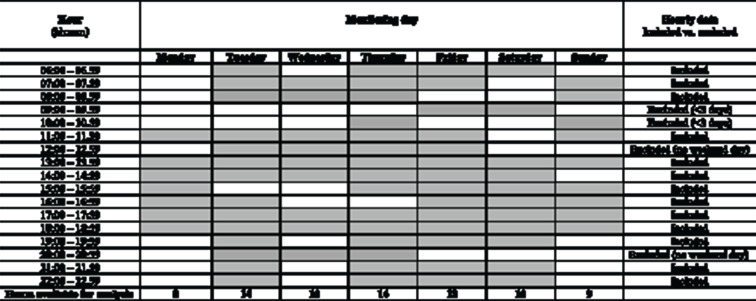
Extraction of hourly data: illustration of valid-wear criteria for one participant. 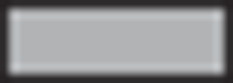
 Indicates valid hour (ie, 60 min of movement data recorded). 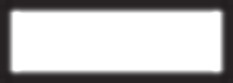
 Indicates invalid hour (ie, <60 min of movement data recorded).

Data were downloaded from the GT3X and analysed using the Actilife software (Version 6.2). Non-wear time was determined by identifying strings of consecutive zero counts recorded by the accelerometer for ≥60 min, allowing for 2 min of counts <100.^27^ Following exclusion of non-wear periods, accelerometer data were analysed on an hour-by-hour basis for all waking hours (ie, 08:00–22:59).^2^[2]8:00–22:59 was a generalised timeframe applied to all data based on all participants graphed data and non-wear periods—that is, 50% of participants (n = 49) were consistently awake and wearing the accelerometer between 8:00 and 23:00, ≥3 days of the week. Before 8:00, only 25% of participants wore the accelerometer for ≥3 days. Time filters were used to separate hourly data (eg, 08:00–08:59, 09:00–09:59, etc) and total wear time within each hour time frame was computed. Participant’s hourly data were retained for inclusion in the final analysis (ie, constituted a ‘valid-wear hour’), where a full 60 min of wear time were recorded within that hour (ie, 60 min/hour), for at least 3 measurement days (including a weekend day).^3^[3]Studies indicate levels of activity differ across week versus weekend days. It is recommended that ≥1 weekend day should be included in analysis, to ensure reliable estimates of habitual PA and sedentary time.
^26 27^ As an example, for the hours 8:00–9:00 to be included for a particular participant, a full 60-min wear time would be needed to be recorded between 8:00 and 9:00 for at least 3 days (eg, Thursday, Friday and Saturday (weekend day), see figure 1 for an illustrative example of data cleaning).

For valid hourly accelerometer data, cut-points were used to compute min/hour spent, sedentary (<100 cpm), and engaged in light-intensity PA (100–2019 cpm) and moderate-to-vigorous-intensity PA (≥2020 cpm).^27^ Sedentary time accumulated in bouts ≥20 min was also determined for each hour (ie, ≥20 consecutive minutes at  < 100 cpm), and the average sedentary bout length within each hour was calculated (min/bout).^11^^4^[4]Accelerometer data processing methods (non-wear criteria and accelerometer cut-points) were selected to facilitate comparisons with the majority of previous RA accelerometer studies and epidemiological research in non-RA populations, in which these criteria have been applied. Hourly accelerometer data were subsequently grouped to represent morning (08:00–11:59), afternoon (12:00–17.59) and evening time periods (18:00–22:59), and data was averaged across the hours within these intervals to quantify sedentary time, light-intensity and moderate-to-vigorous-intensity PA during each time period (eg, morning sedentary time = sedentary time (min) accumulated in hours (08:00 to 08:59 + 09:00 to 9:59 + 10:00 to 10:59 + 11:00 to 11:59) **÷ **4). The approach of dividing the day into morning, afternoon and evening segments to examine diurnal patterns of sedentary time and PA is consistent with approaches used in previous research investigating time-use patterns of sedentary time and PA among older adults.^17 18^

Participant data were only included to estimate morning, afternoon and evening activity, if *all hours* within the corresponding time frame were deemed as ‘valid-wear hours’. For example, to be included in calculations to estimate *morning* sedentary time and PA, participants were required to record valid-wear hours (ie, a full = 60 min of wear time per hour), for *all* of the hours within this time frame (ie, 08:00 to 08:59 + 09:00 to 9:59 + 10:00 to 10:59 + 11:00 to 11:59 = total 240 min). For morning, afternoon and evening, participants were therefore required to have recorded 240, 360 and 300 min of wear time, respectively. To permit relative comparisons between morning, afternoon and evening sedentary time and PA, data are presented as the average min/hour for each activity, for each of the three time periods. In regard to valid *daily* wear time (total min/day of valid accelerometer data), all participants included in the current analysis recorded an average of 13.4 hours/day valid-wear time, which is above the 10 hours/day recommended to provide reliable estimates of sedentary time.^27^

### Preliminary analysis

Descriptive statistics were computed to characterise sedentary time and PA for each hour of the day as well as combined morning, afternoon and evening time periods, using only those participants providing valid-wear data within each hour or for complete morning (valid wear = 240 min, n = 41), afternoon (valid wear = 360 min, n = 64) and evening (valid wear = 300 min, n = 45) time periods, respectively. Following this, participants with valid data for at least two time periods per day (eg, morning and evening, or morning and afternoon) were retained for inclusion in the main statistical analysis (n = 52). Of these participants, a further n = 11 were excluded due to missing QRISK data. The final sample available for analysis was therefore n = 41.

Prior to primary statistical analysis, χ² tests and one-way analyses of variance (ANOVA) were conducted to determine significant differences in demographics, RA characteristics and CVD risk factors, between participants included in the main PARA study at baseline (n = 97 of 115, who were provided with an accelerometer) versus those participants included in the current analysis (ie, n = 41, included on the basis of hourly accelerometer and available QRISK data). In addition, to elucidate any sample bias resulting from exclusion on the basis of hourly accelerometer data, one-way ANOVAs were conducted to compare daily accelerometer wear time as well as daily sedentary time, light-intensity and moderate-to-vigorous-intensity PA (min/day) between participants in this secondary analysis versus those providing valid *daily* accelerometer in the PARA study at baseline (n = 61, see Fenton *et al* (2017) for the original publication of this data).^6^ These analyses revealed that participants included in the current study were not significantly different to those recruited to the larger PARA study in regard to any of the reported characteristics (age, gender, ethnicity, height, weight, RA characteristics, CVD risk factors or accelerometer-assessed activity behaviours). Results of these comparative analyses are provided as online supplementary data (online supplementary file 1).

10.1136/rmdopen-2020-001216.supp1 Supplementary data

### Statistical analysis

Mixed linear modelling (MLM) was used to explore within- and between-participant changes during the day in sedentary time, sedentary bout length, light-intensity PA and moderate-to-vigorous intensity PA separately. This approach was chosen as it does not assume sphericity of the data, can process non-normally distributed data and deal with occasional missing data.^28^ The within-subject level predictor was time (centred by assigning 0 to morning) and the between-person predictor was mean centred CVD risk (QRISK). Sex (women = 0) and mean centred age were entered as level 2 covariates in all analyses.^5^[5]Additional analyses were conducted with employment status as a covariate (employed full time/part time vs unemployed (eg, homemaker, retired, studying)). These analyses revealed no significant effect of employment status in any of the analyses and are therefore not reported. Potential differences in diurnal patterns with varying levels of CVD risk were explored using mean centred CVD risk by time interactions. All analyses were repeated with mean centred disease duration, functional ability (HAQ) and disease severity (ESR) entered as control variables. It is worth noting that CVD risk by time interaction reflects whether the rate of change from morning to either afternoon or evening is similar for varying levels of CVD risk for a female of average age.

## RESULTS

Characteristics of participants included in the main statistical analysis are reported in table 1 (n = 41). Participants were largely women and Caucasian, with moderate disease activity and low to moderate disability. Overall, participants demonstrated a QRISK score indicative of the need for further intervention to lower CVD risk (ie, ≥10%). On average, participants spent approximately 66% of their waking day sedentary, and 32% of their day engaged in light-intensity PA.

**Table 1:** Participant characteristics

	Mean ± SD n = 41	Range (min–max)
Age (years)	58 ± 11	32–74
Gender (% women)	69%	
Ethnicity (% Caucasian)	85%	
Height (cm)	166.1 ± 9.2	151.0–195.0
Weight (kg)	77.1 ± 17.1	51.0–121.7
RA characteristics		
Disease activity (DAS28)	3.18 ± 1.75	0.00–6.28
Erythrocyte sedimentation rate (mmHrs)	16.5 ± 15.8	2.0–69.0
Functional disability (HAQ)	1.67 ± 0.56	1.00–3.00
Disease duration (years)	7.2 ± 8.7	1–37
Morning stiffness (min/day)	35 ± 41	0–180
*Current treatment*		
Anti-TNF (% yes)	10%	
DMARDS (% yes)	56%	
NSAIDS (% yes)	29%	
Analgesics (% yes)	24%	
*CVD risk factors*		
Total cholesterol (mmol/L)	5.0 ± 0.9	3.3–6.9
HDL cholesterol (mmol/L)	1.4 ± 0.4	0.8–2.5
Systolic blood pressure (mm Hg)	136 ± 17	99–181
Diastolic blood pressire (mm Hg)	81 ± 8	67–99
BMI (kg/m^2^)	27.8±5.5	19.7–42.0
Smoker (% current smokers)	7%	
Diabetes (% yes)	7%	
QRISK (%)	15.8 ± 11.9	0.2–48.0
*Activity behaviour*		
Sedentary time (min/day)	514.0 ± 65.6	350–672
Light-intensity PA (min/day)	257.9 ± 67.8	121–423
Moderate-to-vigorous PA (min/day)	18.0 ± 17.2	0.0–76
Sedentary bout length (≥20 min) (min/bout)	31.1 ± 2.3	27–38
Valid wear time (min/day)	789.9 ± 41.8	698–1467

The HAQ typically uses a response scale from 0 (without any difficulty) to 3 (unable to do). In the PARA study, the HAQ was scored on response scale starting at 1 (without any difficulty) to 4 (unable to do).

One participant who did not provide valid *daily* accelerometer data at baseline (excluded as an outlier) was included in the current secondary analysis, as their *hourly* data was considered valid. However, this participant was excluded for the purpose producing descriptive statistics to indicate *daily* estimates of behaviour.

Anti-TNF, anti Tumor Necrosis Factor; BMI, body mass index; CVD, cardiovascular disease risk; DAS28, Disease Activity Score-28; DMARDS, Disease-Modifying Anti-Rheumatic Drugs; HAQ, Health Assessment Questionnaire; HDL, high-density lipoprotein; NSAIDS, non-steroidal anti-inflammatory drugs; PA, physical activity.

### Diurnal patterns of sedentary time and physical activity

Diurnal patterns of sedentary time for morning, evening and afternoon periods are reported in table 2. MLM analyses revealed that participants were significantly more sedentary and less physically active during the evening period, compared to the morning and the afternoon periods. These differences were evident when sedentary time was assessed according to total minutes of sedentary time as well as sedentary bout length. Similarly, lower levels of both light-intensity PA and moderate-to-vigorous-intensity PA were found in the evening period, relative to the morning and afternoon periods. Age was not significantly associated with the diurnal patterns in sedentary behaviour or PA, and no sex differences were reported with the exception of sedentary time, which was marginally higher in women compared to men (p* *= .05).

**Table 2:** Estimated means ± SE for sedentary behaviour and physical activity during the morning, afternoon and evening periods

Time of day	Sedentary time (min/hour)	Light-intensity PA (min/hour)	Moderate-to-vigorousintensity PA (min/hour)	Sedentary bout length (≥20 min)
*Morning* (08:00–11:59)	38.04 ± 1.08	20.13 ± 0.98	1.86 ± 0.31	24.00 ± 1.23
*Afternoon* (12:00–17:59)	39.74 ± 1.01	19.21 ± 0.91	1.07 ± 0.29*	26.22 ± 1.24
*Evening* (18:00–22:59)	47.80 ± 1.16*^,^**	11.97 ± 1.04*^,^**	0.25 ± 0.32*^,^**	29.30 ± 1.13*^,^**

* Significantly different from morning, p < .05.

** Significantly different from afternoon, p < .05.

Values are min/hour and min/bout (for sedentary bout length). Data represents the estimated mean for a female of average age and average cardiovascular disease risk. PA, physical activity.

### Estimated 10-year CVD risk and diurnal pattern of sedentary time

Significant time by CVD risk interaction effects revealed that those with a higher CVD risk spent more time sedentary during the afternoon (p* *= .005) and evening (p* *= .02) compared to those with lower CVD risk. No significant interaction was reported between CVD risk and time spent sedentary in the morning. A significant time by CVD risk interaction was also present for sedentary bout durations; those with higher CVD risk had longer duration sedentary bouts in the afternoon compared to those with lower CVD risk. These analyses were repeated controlling for disease duration, functional ability (HAQ) and disease activity (ESR), which did not change the findings.

### Estimated 10-year CVD risk and diurnal pattern of physical activity

Significant time by CVD risk interactions was also reported for light-intensity PA and moderate-to-vigorous-intensity PA. People with increased CVD risk did less light-intensity PA during the afternoon (*p* = .016) and evening (*p* = .031), as well as less moderate-to-vigorous-intensity PA during the afternoon (*p* = .026). No significant interactions were reported between CVD risk and time spent in light-intensity PA in the afternoon or between CVD risk and moderate-to-vigorous-intensity PA in the morning or evening. Repeating these analyses and adjusting for variations in disease duration, functional ability (HAQ) or disease severity (ESR) did not alter these findings.

## DISCUSSION

This study is the first to investigate diurnal patterns of sedentary time among people living with RA and to determine the associations with estimated long-term (10-year) CVD risk. Results indicate that sedentary time is significantly higher, and sedentary bouts are significantly longer during the evening (18:00–23:00), compared to the morning or afternoon in this patient group. Moreover, higher levels of sedentary time during the evening are associated with an increased estimated 10-year risk of CVD, whereas higher levels of light-intensity PA in the evening are linked to lower estimated 10-year CVD risk.

Current findings suggest that interventions targeting evening sedentary time may offer a significant opportunity for intervention among people living with RA and may be particularly valuable in regards to reducing CVD risk. Our results demonstrating levels of sedentary time are highest in the evening are in alignment with existing research in RA that describes a decline in ‘average accelerometer activity counts’ to occur throughout the day.^12 29^ These previous investigations aggregated ‘average activity counts per hour’ across 3-hour time periods, reporting the predominant activity behaviour undertaken in the late morning (9:00–12:00), midday (12:00–15:00) afternoon (15:00–18:00) and evening (18:00–21:00). Results revealed average activity counts declined from approximately 8000 counts/hour in the late morning (or 133 cpm) to 5000 counts/hour in the evening (or 83 cpm). Interpretation of these data using the cut-points applied in the current study suggests that RA patients were predominantly engaged in light-intensity PA in the morning (ie, 100–2019 cpm) and spent most of their time sedentary during the evening (ie, < 100 cpm). However, as these studies did not statistically examine within-person changes in activity behaviours, more conclusive comparisons with current data cannot be made. Additional research is therefore required to further substantiate the conclusion that the evening time period may represent an ideal time for interventions to reduce sedentary behaviour in RA, owing to the high sedentary levels observed during this period.

A consideration of the factors underlying engagement in sedentary behaviours (ie, determinants) will also be critical to consider when developing such interventions. Indeed, while high levels of sedentary behaviour during the evening offer a significant opportunity for intervention, there may be other factors (determinants) that influence whether an individual would engage in interventions to reduce their engagement in sedentary behaviours during the evening. To date, few studies have sought to identify determinants of sedentary behaviour in RA, which—due to the pathophysiology of this disease—are likely to be complex and multifactorial. For example, factors related to the chronobiology of RA may represent salient individual determinants of behaviour, which interact with other individual and environmental factors to influence sedentary time. Research indicates that joint stiffness is most pronounced in the morning among people with RA, an association suggested to be mediated by diurnal rhythms of cytokine levels.^30^ People living with RA also report heightened feelings of fatigue in the afternoon.^31^ These diurnal rhythms may explain both the higher levels of light-intensity PA undertaken in the morning (ie, to alleviate morning stiffness) and the afternoon and evening ‘sedentary windows’ observed in this study (ie, in response to fatigue). Indeed, fatigue and morning stiffness have been identified as two key factors influencing levels of sedentary time engagement among people living with RA.^32^ While we were unable to assess diurnal patterning of biological factors in this study, this represents an important and interesting avenue for future research.

Comparison of diurnal patterns of sedentary time engagement between people living with vs. without RA may provide some insight into the role of RA-specific determinants in this regard. In studies of older adults living without RA (aged  ≥ 65 years), similar patterns of sedentary time engagement have been observed, whereby sedentary time is lower during the morning (before 12:00) and peaks in the evening (eg, after 17: 00–19:00). However, in observing absolute levels of sedentary time, morning sedentary time was reported to be higher among RA patients in our study, compared to other studies of older adults (consistently + 8 min/hour in RA).^16–18^ Moreover, in one study that examined differences in sedentary time among older adults in the morning (7:00–12:00) vs. the afternoon (12:00–17:00), sedentary time estimates increased from the morning (30 min/hour) to the afternoon (39.8 min/hour),^17^ where sedentary time among our population of RA patients was largely unchanged (38.0 and 39.7 min/hour in the morning and afternoon, respectively). Such comparisons may indicate that indeed, RA-specific factors are at play (eg, morning stiffness linked to inflammatory mechanisms) and are impacting diurnal patterns of sedentary time in this patient group. As such, additional research directed at understanding, and subsequently harnessing the concept of chronobiology to understand time-use patterns of behaviour among people with RA, may help to develop and deliver more optimally timed interventions and more effective patient care for this population.

We must also be cognisant of non-RA-related factors when considering avenues for intervention. For example, in a recent qualitative study, RA patients reported that while symptoms such as pain and fatigue sometimes influenced their activity, their decision to engage in sedentary behaviour could also have nothing to do with their RA, and instead reflected a way of living independent of their disease.^32^ Social relations were described as to contributing towards increased sedentary behaviour (eg, coffee mornings, film/movie night), and a lack of motivation to ‘move’ was also reported to influence levels of sedentary time engagement in this patient group. As such, in developing interventions to reduce sedentary behaviour in RA, we must work not only work to understand patterns of sedentary time accumulation (ie, identify ‘sedentary windows’ of opportunity) but also to identify salient RA-related and non-RA-related determinants of sedentary behaviour that can be targeted by such interventions, in order to effectively encourage behavioural change.^33^

Current results suggest that a feasible intervention approach may be to support individuals to reduce their sedentary time, by engaging in regular, short periods of light-intensity PA throughout the day (eg, standing or walking ‘sedentary breaks’). Indeed, simultaneous investigation of light-intensity PA behaviour and sedentary time indicated opposite diurnal patterns of engagement. This mirroring of behaviour adds further impetus to the proposition that encouraging people living with RA to engage in light-intensity PA may result in reductions in sedentary time (ie, via displacement).^6^ This approach may prove particularly effective for interventions targeting the evening ‘sedentary window’, which—as current data indicates—is the most sedentary period of the day and is characterised by more prolonged, uninterrupted sedentary bouts.

In our previously published analysis, we demonstrate that higher daily sedentary time (including bouts per day ≥20 min) was positively associated with increased 10-year CVD risk estimated using the QRISK2, with the reverse negative association observed for light-intensity PA.^6^ The current investigation extends these analyses, to reveal that individuals with RA who accumulate the most sedentary time (overall and uninterrupted bouts ≥20 min), and the least light-intensity PA during the afternoon and evening, appear to be at the greatest risk for developing CVD in the subsequent 10 years. Thus, as well as representing the best opportunity for encouraging behavioural change, interventions targeting evening sedentary time in particular, may be particularly valuable in regard to their potential to reduce CVD risk in RA.

Potential mechanisms that may be responsible for the association between sedentary time and increased CVD risk include decreased lipoprotein lipase activity and compromised vascular function.^14^ ^34–36^ However, it is also possible that the increased CVD risk associated with higher sedentary time is influenced by other context-specific behaviours that occur in parallel to sedentary pursuits.^14 37 38^ This may offer one explanation as to why higher sedentary time accumulated specifically in the afternoon/evening may be associated with increased CVD risk. For example, energy intake and consumption of high energy ‘snack foods’ are reported to be significantly greater among individuals who engage in the most television viewing—a prominent leisure time sedentary behaviour.^37 39^ Moreover, epidemiological evidence suggests that TV time energy intake mediates the relationship between TV viewing and abdominal obesity in young adults,^40^ and individuals who jointly report higher television viewing and increased snack food consumption are at increased risk of developing metabolic syndrome and its individual components (eg, obesity).^41^ Thus, additional research is necessary to determine whether it is the deleterious physiological consequences of sedentary time per se that contribute towards heightened CVD risk, or whether this adverse association is a result of other ‘unhealthy’ behaviours engaged in while sedentary, which contribute to the CVD risk factor burden.

A limitation of this study is a lack of control group of healthy adults, with which to compare diurnal patterns of sedentary behaviour observed among this group of RA patients. However, we have drawn comparisons with other studies that have examined diurnal patterns of sedentary behaviour among older adults, to provide some insight into how levels and patterns of sedentary time may be compared between RA and non-RA populations. The study sample was also drawn from a larger RCT examining the efficacy of intervention to promote PA in RA.^19^ Here, we conducted a secondary analysis using baseline data to answer the current research questions. As participants volunteered to take part in the RCT, their levels of sedentary behaviour and PA may not reflect those typical of the general RA population. Future research should therefore be conducted using an independent sample of RA patients and age-, sex- and BMI -matched healthy controls.

Additional limitations of this study include a reliance on cross-sectional data and reduced sample size available for statistical analysis. The cross-sectional nature of the data means that we cannot determine causal associations between sedentary time and long-term CVD risk and rule out reverse causality (ie, that people with a higher CVD risk may be more sedentary). Experimental studies that aim to reduce sedentary time and measure corresponding changes in CVD risk factors and long-term CVD outcomes are therefore required to confirm the value of sedentary behaviour interventions in RA. The reduced sample size was as a result of employing a strict analytical approach requiring complete ‘valid-data’ across individual hours (ie, full 60-min wear/hour), and subsequently morning, afternoon and evening time periods, for inclusion in the analysis. This method was adopted to reduce bias at the most granular level of the data, feeding into the primary variable of interest in regard to our research question (ie, morning, afternoon and evening sedentary time and PA).

With this approach, participants included in analysis recorded an average of 13.4 hours/day valid-wear time, which is above 10 hours/day recommended to provide reliable estimates of sedentary time.^19^ To maximise sample size, we (1) employed a generalised time frame to participants’ accelerometer data (08:00–22:59), based on visual inspection of participants graphed data and typical sleeping/waking times, (2) included participants in analysis who recorded complete valid data for ≥2 (out of a possible 3) time periods and (3) employed MLM as our primary analytical approach. MLM is able to deal with occasional missing data (eg, either morning or afternoon or evening data in this study), and thus enabled investigation of within- and between-participant changes in sedentary behaviour and PA during the day in this study, to begin to answer important, novel questions regarding the chronological succession of sedentary time in RA. Still, future research using larger samples with more complete data profiles are required to confirm the findings reported in this study, as missing accelerometer data occurring across some hours may indeed limit the inferences that can be made.

General limitations of using accelerometry to measure PA and sedentary time in this study should also be acknowledged. While accelerometers offer a more objective assessment of PA and sedentary time relative to self-report, accelerometers have not been specifically validated for measurement of PA and sedentary among people living with RA. That is, the algorithms applied to quantify PA intensities and sedentary time in this study were developed in studies of healthy adults, and therefore do not consider the unique physiology of people living with RA (eg, a higher resting metabolic rate). The use of accelerometers in this study also means that the definition of sedentary behaviour employed considers only energy expenditure (ie, ≤1.5 METs) and not the posture in which low-energy behaviours occurred.^15 42^ Together, these limitations of accelerometery highlight a requirement for future RA research that employs multiple methods (eg, self-report, accelerometry, posture sensors) that have been validated for use specifically among people living with RA, in order to build a comprehensive picture of what, where, when and how sedentary behaviour occurs in this patient group, prior to intervention.^15^

In conclusion, results suggest that people living with RA are significantly more sedentary during the evening (after 18:00) period, when compared to the morning (8:00–11:59) and the afternoon (12:00–17:59). In addition, higher sedentary time and more prolonged (uninterrupted) sedentariness during the evening are linked to increased estimated 10-year risk of CVD in this patient group. Thus, we provide preliminary data to suggest that the evening period may represent a ‘sedentary window’ for behavioural intervention and associated improvements in CVD risk among people living with RA. Due to inverse patterns of engagement, replacing or interrupting sedentary time with light-intensity PA (eg, via encouraging sedentary breaks) may offer an effective intervention approach for this population. Identifying RA-related and non-RA-related factors (eg, symptoms, motivation) that may influence engagement in sedentary behaviour is an important avenue for future research that will be critical in optimising such interventions for this patient group.

**Key messages**

- Sedentary behaviour (‘too much sitting’) demonstrates adverse associations with cardiovascular disease (CVD) risk, with emerging evidence of deleterious consequences for cardiovascular health in rheumatoid arthritis (RA).
- This study is the first to examine diurnal patterns of sedentary time in RA and associations with CVD risk.
- Results revealed sedentary time was highest in the evening among people living with RA and that individuals with a higher CVD risk also spent more time sedentary during the evening, compared to those with lower CVD risk.
- Results suggest that the evening may represent a critical time period or ‘sedentary window’ of opportunity for behavioural intervention, and associated improvements in CVD risk among people living with RA.
