# Supplementary material for: Diurnal patterns of sedentary time in rheumatoid arthritis: associations with cardiovascular disease risk
Source: RMD Open. 2020 Jul 14;6(2):e001216. doi: 10.1136/rmdopen-2020-001216 (PMC7425187; doi:10.1136/rmdopen-2020-001216)
Supplement: Supplementary data [file rmdopen-2020-001216s001.pdf]

Supplementary Table 1. Characteristics of participants included secondary analysis (diurnal patterns) vs. those included in the main PARA study at baseline

	**Mean ± SD**<br>**n = 41**	**Mean ± SD**<br>**n = 97**
Age (years)	58 ± 11	54 ± 12
Gender (% female)	66%	69%
Ethnicity (% Caucasian)	85%	84%
Height (cm)	166.1 ± 9.2	166.8 ± 8.7
Weight (kg)	77.1 ± 17.1	77.9 ± 18.3
**RA characteristics**		
Disease activity (DAS28)	3.18 ± 1.75	3.53 ± 1.43
Erythrocyte Sedimentation Rate (mmHrs)	16.5 ± 15.8	15.4 ± 14.7
Disease severity (HAQ)	1.67 ± 0.56	1.73 ± 0.60
Disease duration (years)	7.2 ± 8.7	7.4 ± 8.4
Morning stiffness (minutes/day)	59.6 ± 159.7	55.3 ± 116.1
Current treatment		
Anti-TNF (% yes)	10%	14%
DMARDS (% yes)	56%	52%
NSAIDS (% yes)	29%	31%
Analgesics (% yes)	34%	39%
**CVD risk factors**		
Total cholesterol (mmol/L)	5.0 ± 0.9	5.1 ± 1.0
HDL cholesterol (mmol/L)	1.4 ± 0.4	1.5 ± 0.4
Systolic blood pressure (mmHg)	136 ± 17	133 ± 17
Diastolic blood pressire (mmHg)	81 ± 8	81 ± 9
Body-mass-index (kg/m^2)	27.8 ± 5.5	27.9 ± 6.1
Smoker (% current smokers)	7%	9%
Diabetes (% yes)	7%	6%
QRISK® (%)	15.8 ± 11.9	14.2 ± 12.4
**Activity behaviour**		
Sedentary time (min/day)	514.0 ± 65.6	497.4 ± 68.3
Light PA (min/day)	257.9 ± 67.8	269.4 ± 69.4
Moderate-to-vigorous PA (min/day)	18.0 ± 17.2	18.0 ± 17.3
Sedentary bout length (≥ 20min) (min/bout)	31.1 ± 2.3	30.1 ± 2.5
Valid wear time (min/day)	789.9 ± 41.8	784.8 ± 45.5

Note: Significant differences between samples are indicated as * p <.01, ** p <.05. For activity behavior variables, comparative analysis was conducted on only those participants providing valid accelerometer at baseline (i.e., ≥10 hours day, on ≥4 days, including a weekend day, n = 61). One participant who did not provide valid *daily* accelerometer data at baseline (excluded as an outlier), was included in the current secondary analysis, as their hourly data was considered valid. However, this participant was excluded for the purpose producing descriptive statistics to indicate *daily* estimates of behavior, and were therefore also excluded from comparative analysis which used daily activity behavior values.
